# Ireland

**Published:** 1914-09-05

**Authors:** 


### IRELAND.

*University of Dublin (Trinity College).*—The Medical School of Trinity College, or School of Physic in Ireland, was established in 1711. All classes are open to external students as well as to students of the University, provided that they have passed an examination in Arts recognised by the General Medical Council. Women students are admitted to the classes and to the University degrees. The winter courses begin on October 1. Candidates for the degrees in Medicine (M.B.), Surgery (B.Ch.), and Midwifery (B.A.O.) must be of B.A. standing, and must have spent at least five academic years, reckoned from the date of Matriculation, in the study of Medicine. The Arts course may be taken concurrently with the Medical course, and the B.A. degree need not be taken before the final medical examinations, but the medical degrees are not conferred without the Arts degree. The M.D. degree is awarded to graduates in Medicine of M.A. standing who have read an approved thesis publicly before the Regius Professor of Physic. Special classes are held for the Diploma in Public Health.

*Royal College of Surgeons in Ireland (the Schools of Surgery).*—These schools are attached by charter to the Royal College of Surgeons in Ireland. They are carried on within the college buildings, and are specially subject to the supervision and control of the Council, which is empowered to appoint and remove the professors, and to regulate the methods of teaching pursued. The buildings have been reconstructed, the capacity of the dissecting-room nearly trebled, and special pathological, bacteriological, public health, chemical, and pharmaceutical laboratories fitted with the most approved appliances, in order that students may have the advantage of the most modern methods of instruction. There are special rooms set apart for lady students. The medical students' guide will be forwarded post free on application to the Registrar, Royal College of Surgeons, Dublin.

*The Queen's University of Belfast.*—This University provides all the classes required for a complete medical curriculum. The University contains laboratories in connection with the departments of biology, chemistry, physiology, pathology, anatomy, physics and materia medica. Women are eligible as students. Clinical instruction is given at the Royal Victoria Hospital, which was rebuilt a few years ago and has 300 beds, and at the Mater Infirmorum Hospital, which has 150 beds. Other hospitals open to the students of the University are: The Maternity Hospital, the Ulster Hospital for Women and Children, the Hospital for Sick Children, the Ophthalmic Hospital, the Benn Ulster Eye, Ear, and Throat Hospital, the Union Infirmary and Fever Hospital, the Fever Hospital, Purdysburn, and the District Lunatic Asylum, the Samaritan Hospital, the Forster Green Hospital for Consumption and Chest Diseases, and the Belfast and Ulster Hospital for Diseases of the Skin. The cost of the curriculum intended for students proceeding to the degrees of the Queen's University of Belfast is, approximately, £105. This includes examination fees and a perpetual ticket for attendance at the Royal Victoria Hospital or the Mater Infirmorum Hospital, but not fees for the special hospitals. The course for the Conjoint Board costs about the same amount. A pamphlet containing full information regarding the new regulations for courses, fees, etc., can be had free of cost on application to the Secretary, Queen's University, Belfast.

*University College, Cork.*—Students can attend courses which meet the requirements of the National University of Ireland, the Conjoint Boards of London, Edinburgh, or Dublin, and other examining bodies. Clinical instruction is given at the North and South Infirmaries, each of which contains 100 beds, at the District Hospital, which contains 1,200 beds, and at other hospitals. The college is a constituent college of the National University of Ireland, and holds its own examinations in all subjects required for the degrees of that University. Thirty-six Entrance and County Council Scholarships, varying from £50 to £20, which may be held in any Faculty, are offered for competition. There are, in addition, twelve scholarships of about £30 each offered for competition to medical students of the second, third, fourth, and fifth years. The Blayney Scholarship, of about £32, is awarded to the candidate who, being of the prescribed standing, obtains highest marks with honours at the National University examination for the M.B., B.Ch., B.A.O. degrees, taken as a whole. Further information can be had on application to the Registrar, University College, Cork.

*University College, Galway.*—In addition to various laboratories, a dissecting-room, and anatomical lecture theatre, the College contains a large natural history and geological museum, as well as an extensive library for the students' use. New chemical and pathological laboratories are in course of construction. There are six junior scholarships of the annual value of £25 each specially reserved for students in medicine, and awarded on the results of the University examination of the corresponding year. In addition, medical students at entrance are eligible to compete at the General Entrance Scholarship examination, and to hold the scholarship, if successful, in the Faculty of Medicine. Clinical instruction is given in the Galway Hospital and in the Galway Union and Fever Hospitals. Full information as to courses of lectures, scholarships, and class fees can be obtained from the Registrar, University College, Galway.

*The Medical School of the Catholic University, Ireland, Dublin.**—Founded in 1855, it is now the largest medical school in Ireland. It prepares students specially for the examinations of the Royal University and the Conjoint Colleges of Ireland and Edinburgh, but its lectures and practical courses are recognised by all the licensing bodies in Great Britain and Ireland. In addition to the ordinary medical examinations it prepares students for the D.P.H. and for the various higher University examinations in pathology, physiology, chemistry, etc. Six exhibitions and numerous gold and silver medals are offered annually for competition.

* No returns received.

